# Collective Enhancements on Thermal-Electrical and Mechanical Properties of Graphite-Based Composite Bipolar Plates through the Coupled Manipulations of Molding and Impregnation Pressures

**DOI:** 10.3390/membranes12020222

**Published:** 2022-02-15

**Authors:** Xueliang Wang, Zhiguo Qu, Haitao Yang, Guobin Zhang, Yichong Zhang, Chaofan Liu

**Affiliations:** 1 Moe Key Laboratory of Thermo-Fluid Science and Engineering, Energy and Power Engineering School, Xi’an Jiaotong University, Xi’an 710049, China; xlwang082@mail.xjtu.edu.cn (X.W.); yanghaitao13@stu.xjtu.edu.cn (H.Y.); zhangguobin@xjtu.edu.cn (G.Z.); 2 Shanghai Sinofuelcell Co., Ltd., Shanghai 201499, China; liucf@sl-power.com

**Keywords:** bipolar plates, graphite-based composite, molding pressure, impregnation pressure, interfacial contact resistance, thermal conductivity, mechanical property

## Abstract

The performance and durability of proton exchange fuel cells (PEMFCs) are greatly affected by the bipolar plate (BP). In this paper, the thermal and electrical conductivities and mechanical property of graphite filled with resin composite BPs were collectively enhanced through the effectively coupled manipulations of molding pressure and impregnation pressure. The microstructures show that the resin tends to distribute at the top region of the rib under high impregnation pressure. The thermal and electrical conductivities of the pure expanded graphite BP is well reserved in the composite BPs under high molding pressure, which can facilitate the heat transfer and electron conduction in the PEMFCs. The relative density and compressive strength of composite BPs were greatly enhanced by the impregnation of resin compared to the expanded graphite under high molding pressure without the impregnation of resin (HU-BP). The maximum thermal conductivity, compressive strength, and minimum interfacial contact resistance (ICR) are collectively achieved in the HL-BP. The enhanced thermal-electrical and mechanical properties could be mainly attributed to the well-reserved continuous networks of graphite in the composite BPs. The findings in this paper are expected to synergetically improve the thermal, electrical, and mechanical properties of composite BPs through coupled manipulations of the molding and impregnation pressures, which in turn enhances the power density and durability of PEMFCs.

## 1. Introduction

Proton exchange membrane fuel cells (PEMFCs) are considered one of the most competitive energy-utilization devices for carbon emission reduction because of their high power density, low operating temperature, and pollution-free reaction products. As a key component in PEM fuel cells, the bipolar plates (BPs), where the flow fields are pressed, account for 40–80% of the stack weight and 30–40% of the stack cost [1,2]. Furthermore, optimization in BPs will promote ~20% in the stack power density (6.0–9.0 kW L^−1^) [3]. Consequently, there is great potential to improve PEMFC performance and durability through enhancing the performance of BPs.

The specific function of BPs in the PEMFC stack can be described as follows: the reactant gas is fed in and then distributed in the cell through the flow field pressed in the BPs [4,5]. Meanwhile, the electrons generated in the reaction are conducted to the external circuit via the BPs. Consequently, the BP functions can be enhanced from two aspects, one is the electron/heat conduction (material selection), and another is gas/liquid transport (flow field structure design). The electrical and thermal conductivities, mechanical strength, and corrosion resistance of BP are mainly determined by the material, while the reactant gas diffusion and resultant liquid water transport capacities are controlled by the flow field structure.

Currently, metallic BPs such as stainless steel [6,7], aluminum [8,9], titanium [10,11], carbon steel [12,13], and nickel alloys [14] are developed and applied as BPs in PEMFCs due to their high electrical conductivity, high mechanical strength, low gas permeability, ultra-thin shape, and low manufacturing costs. Nevertheless, these metallic BPs suffer from corrosion problems and require further surface protective coating processes. Compared with metallic BPs, carbon-based materials such as graphite materials are considered promising candidates in the future due to their high chemical-corrosion resistance, lightweight, and low cost. However, the intrinsic brittleness, low mechanical strength, and high gas permeability limit the application of pure graphite materials in PEMFCs. Fillers such as resin and polymer are added to the graphite matrix to achieve a balance between the mechanical strength, gas tightness, and electrical/thermal conductivities in graphite-based composite materials.

Strategies have been developed to enhance the mechanical and electrical properties through tailoring the type of filler or graphite shape, the filler content, and so on. Kang et al. [15] studied the effects of filler type on the performance of fuel cells and found that resin composite filled with graphite flakes shows higher electrical conductivity and lower flexural strength than that filled by graphite lumps. The graphite flakes act as an effective conduction network and facilitate the conduction of electrons. Alo et al. [16] studied the electrical and mechanical properties of polypropylene/epoxy-filled graphite/carbon black composite. The electrical conductivity increases from 49 to 90 S cm^−1^ in the in-plane (IP) direction and 0.37 to 9.34 S cm^−1^ in the through-plane (TP) direction when the filler (graphite/carbon black) content increases from 50% to 80%. Roncaglia et al. [17] investigated the effects of different methods, i.e., the wet and dry mixing on the properties of composite materials. The results show that the electrical conductivity is 100% higher for the composite from the wet mixing method than that from the dry mixing method. Li et al. [18] studied the combination of vacuum resin impregnation and hot press methods for fabricating the expanded graphite filled with resin composite material. The performance of fuel cells assembled with graphite-resin composite BP is superior to that of the graphite BP. Apart from the electrical and mechanical properties of BPs, the thickness of BP is also a key target parameter that affects the power density of PEMFC stacks. Kang et al. [19] developed an ultralight and thin epoxy-carbon/graphite composite BP, in which the thickness is only 0.6 mm with improved electrical conductivity (in-plane, 172 S cm^−1^) and moldability. The distribution and uniformity of fillers in the matrix have a significant impact on the property of composite BP materials, while the distribution of resin in the graphite matrix is mainly affected by the molding and impregnation pressures. Nevertheless, the coupled effects of molding pressure and impregnation pressure on the mechanical and electrical/thermal conductivity remains unclear and needs to be further explored and clarified.

In this paper, to optimize the electrical-thermal and mechanical properties of graphite matrix filled with thermoset resin composite BPs, the effects of molding and impregnation pressures on the microstructure evolution and property of the composite BPs were investigated. The thermal and electrical conductivities and mechanical property of composite BPs were collectively enhanced through coupled optimization of molding pressure and impregnation pressure. The findings in this paper are expected to help design graphite-based composite BPs with collectively enhanced thermo-electrical-mechanical properties through the effective tailoring of molding and impregnation pressures, which can further enhance the power performance and durability of PEMFCs.

## 2. Materials and Methods

### 2.1. Fabrication of BPs

**Figure 1:**
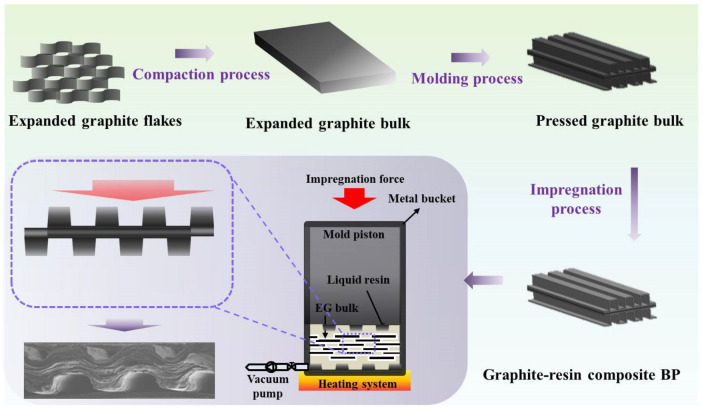
Schematic illustrations of the fabrication process of composite BPs following the compaction process, molding process, and impregnation process.

In this work, the expanded graphite plate, obtained from graphite flakes being molding pressed under specific pressures, is used as a matrix material for the impregnation of thermosetting resin. The thermoset resin (1.08 g/cm^3^) is composed with isobornyl methacrylate (C_14_H_22_O_2_, 95 wt.%, Aladdin, Shanghai, China ), which is the main ingredient mixed with electrical and thermal conductive fillers. Figure 1 shows the schematic fabrication process of graphite-based composite BPs, which is similar to the process described in previous work [20] and mainly includes three steps. The first step is the compaction of expanded graphite flakes into expanded graphite plates under specific pressure. The overall size of the obtained graphite plate is 500 × 500 × 5 mm, and the corresponding bulk density is ~0.2 g/cm^3^. The second step is the stamp of the classic straight rib-channel flow field using the molding compression process. The area ratio of the rib/channel is 1:1. The third step is the impregnation of thermosetting resin into the expanded graphite matrix. Specifically, as illustrated in the lower-left corner in Figure 1, the graphite plate pressed with flow field is put in the pressure-tight metal bucket assembled with stainless steel mold (liquid resin mixture inside the bucket), in which the vacuum degree is kept below −0.1 MPa. The thermosetting resin solution was pumped into the mold and forced to penetrate the porous graphite plate under specific impregnation pressure. Then, the obtained graphite-resin composite BP is cured at the temperature range of 80–100 °C and further treated by surface treatment.

To evaluate the effects of molding pressure and impregnation pressure on the distribution of resin in the porous graphite matrix and further the thermal-electrical and mechanical properties of the graphite-resin composite BPs. Three different molding pressures, i.e., 15 MPa (high), 10 MPa (medium), and 5 MPa (low), and three different impregnation pressures i.e., 30 MPa (high), 20 MPa (medium), and 10 MPa (low) were used in the fabrication process, as displayed in Table 1. To simplify the experimental process and obtain valid experimental data, the samples from the combination of medium and small pressures were removed. Consequently, five graphene-resin composite BPs, i.e., the HL-BP, HM-BP, HH-BP, MH-BP, LH-BP, were designed and fabricated. For comparison, the pressed expanded graphite plate under high pressure without the impregnation of resin (HU-BP) was also employed in the subsequent analysis.

### 2.2. Characterization of BPs

To evaluate the impact of molding pressure and impregnation pressure on the distribution and impregnation ratio of resin in the expanded graphite matrix, microstructure, and phase composition of the graphite-resin composite BPs were characterized using field emission electron scanning microscope (SEM) Gemini-500, ZEISS, (Oberkochen Germany), X-ray diffraction XRD-7000S, using Cu-K_a_ radiation, X-ray diffraction = 1.54060 Å, Shimadzu (Kyoto, Japan), and a Raman spectrometer HR800, 633 nm, HORIBA ( Gières, France). To evaluate the effect of resin distribution on the thermal-electrical and mechanical properties of the graphite-resin composite BPs, the thermal conductivity, interfacial contact resistance, and compressive strength were tested. Specifically, the thermal conductivity of the graphite-resin composite BPs was obtained using the transient method by testing the thermal diffusion coefficient using Nanoflash LFA447, NETZSCH (Selb, Germany), and the specific heat capacity was obtained using differential scanning calorimeter DSC-2000,TA Instruments, (New Castle, DE, America), and density ZMD-2 (Shanghai RuiFang, China). The in-plane (IP) and through-plane (TP) electrical conductivities and the interfacial contact resistance (ICR) of the graphite-resin composite BPs were tested using the electrical conductivity tester FT-541SJB-341 (Ningbo Ruike, China). The compressive strength of the graphite-resin composite BPs was tested using a universal material testing machine CTM8010 (Shanghai Xieqiang, China).

## 3. Results and Discussions

### 3.1. Microstructure Characterization of BPs

**Figure 2:**
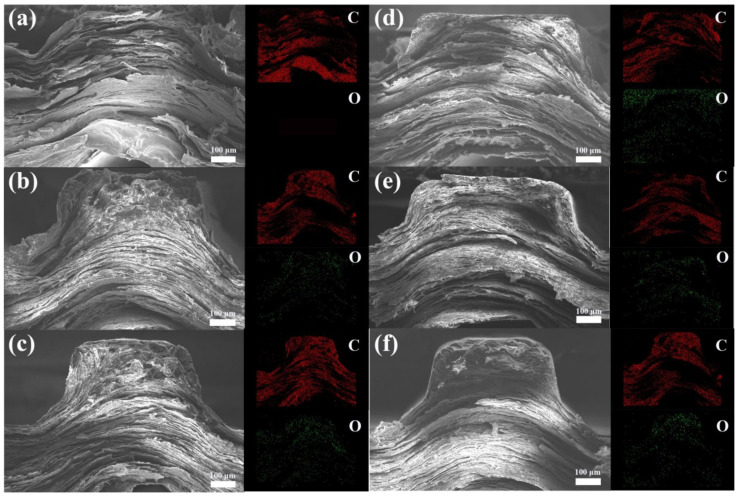
SEM morphology and corresponding EDS map of graphite-resin composite bipolar plates (BPs). (**a**) HU-BP, (**b**) HL-BP, (**c**) HM-BP, (**d**) HH-BP, (**e**) MH-BP, and (**f**) LH-BP.

The cross-section morphology of channel-rib in the graphene-resin composite BP is investigated to evaluate the effect of molding and impregnation pressure on the evolution of composite microstructure. Figure 2a shows the rib of HU-BP with a well-reserved lamellar layered structure, which is out of shape, and obvious gaps are displayed between the graphite sheets, indicating that the moldability of pure expanded graphite matrix is poor. The corresponding energy disperses spectroscopy (EDS) map on the right side of Figure 2a shows that the element in the HU-BBP is almost carbon with invisible oxygen concentration, indicating that the impurities in the expanded graphite were significantly removed during the high-temperature pyrolysis process. The lamellar layered structure could provide a continuous conduction network for heat and electrons, which enhances the thermal and electrical conductivity. Figure 2b shows the morphology of HL-BP with obvious void defects on the top corners of the rib, indicating that the resin is not sufficiently saturated in the graphite matrix under low impregnation pressure. The lamellar layered structure in the HL-BP degrades compared to the HU-BP, which may deteriorate the crystal structure of the graphite matrix and further impact the thermal and electrical conductivities. The corresponding EDS map shows that the majority is carbon with a little oxygen concentration dispersed in the cross-section region. The oxygen element in the HL-BP can be mainly from the penetrated resin as compared to the HU-BP.

Figure 2c shows the morphology of HM-BP with a clear and regular rib shape, indicating that the moldability of HM-BP is greatly improved compared to the HL-BP with the increase of impregnation pressure. The lamellar layered structure of graphite degrades, especially at the top region of the rib. The corresponding EDS map shows that the concentration of oxygen is much higher in the top region of the rib than in the other regions. The significant morphology change in the top region of the rib indicates that the resin tends to disperse in the top region of the rib, and the moldability of the composite BP is greatly improved by impregnating the resin. Figure 2d shows the morphology of HH-BP with a flat fracture surface and dense texture throughout the whole cross-section area. Similar to the EDS map of HM-BP, the oxygen concentration is much higher in the top region of the rib than in the other region. The morphology and EDS map indicate that the porous graphite matrix is sufficiently saturated with resin under high molding and impregnation pressures, and the moldability of composite BP is greatly enhanced with a higher fraction of resin. The composite texture evolves from lamellar layered structure to dense flat bulk under high molding pressure with increasing impregnation pressure.

To evaluate the effect of molding pressure on the microstructure of composite BP, the cross-section SEM morphology of BPs under high impregnation pressure combined with medium and low molding pressures was investigated. Figure 2e shows the morphology of MH-BP with dense texture and flat cross-section surface. The corresponding EDS map shows that the oxygen dispersed uniformly in the whole cross-section region of the rib, which indicates that the resin dispersion uniformity is effectively improved in the porous graphite matrix under medium molding pressure combined with high impregnation pressure. Figure 2f shows the morphology of LH-BP with a laminar layered structure in the cross-section region of the rib except for the dense texture in the top corners of the rib. The corresponding EDS map shows that the oxygen concentration is higher in the top corners than the other regions, which indicates that the resin priors to the top corners of the rib under low molding pressure combined with high impregnation pressure.

It can be concluded from the above microstructure evolutions that the moldability of composite BP is greatly improved with high molding pressure combined with increasing impregnation pressure. Meanwhile, the laminar layered structure of graphite degrades and evolves into dense bulk morphology with increasing molding pressure combined with high impregnation pressure.

**Figure 3:**
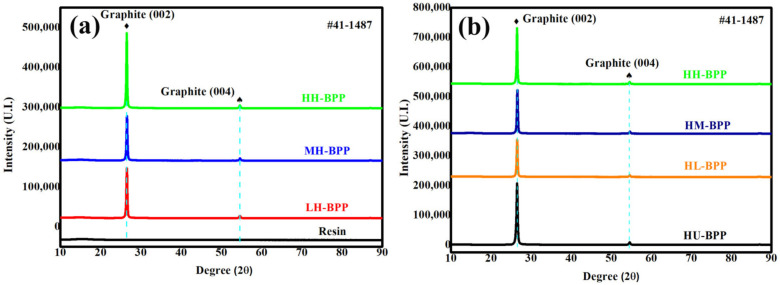
XRD spectra of composite BPs under (**a**) various molding pressures combined with high impregnation pressure and (**b**) high molding pressure combined with various impregnation pressures.

The XRD spectra of composite BPs were tested to evaluate the effect of resin impregnation on the phase composition evolution. Figure 3a shows the XRD pattern of composite BPs under different molding pressure combined with high impregnation pressure and resin mixture. There is no obvious diffraction peak in the resin due to the mixture. In contrast, the diffraction peak of graphite (002) and (004) are displayed in all three composite BPs. Specifically, the intensity of graphite peak (002) increases gradually with the increase of molding pressure, which indicates that increasing the molding pressure could improve the graphite crystal structure. Figure 3b shows the XRD pattern of composite BPs under high molding pressure combined with different impregnation pressure. The intensity of graphite peak (002) is lower for the HL-BP, HM-BP, and HH-BP than the HU-BP, indicating that the impregnation of resin deteriorates the crystal structure of the graphite matrix. The deterioration of graphite crystal structure is considered to impact the thermal and electrical conductivities of composite BPs.

**Figure 4:**
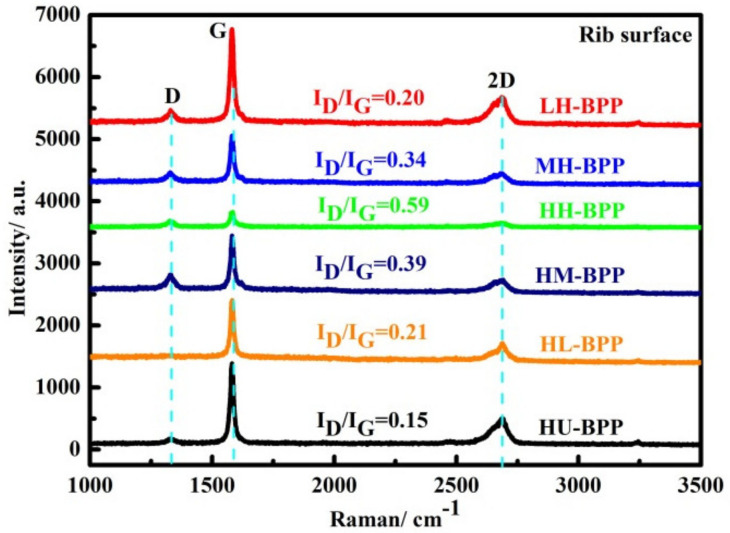
Raman spectra of composite BPs under different combination of molding and impregnation pressures.

The effect of resin impregnation on the crystal structure of layered graphite was further evaluated by testing the Raman spectra of composite BPs (rib surface region). The D (1327 cm^−1^), G (1580 cm^−1^), and the second-order of the D (2D, 2680 cm^−1^) peaks are displayed in all the composite BPs as shown in Figure 4. The intensity of the D peak decreases with increasing molding pressure combined with high impregnation pressure. To quantitatively evaluate the crystal structure evolution of graphite, the intensity ratio of D peak and G peak is calculated and is displayed in Figure 4, and the I_D_/I_G_ increases for composite BPs with increasing molding pressure, as well as with increasing impregnation pressure, and the maximum ratio is displayed in the HH-BP. The higher I_D_/I_G_ ratio in the composite BPs than the HU-BP can be attributed to the crystal structure deterioration of graphite induced by resin impregnation.

### 3.2. Thermal Conductivity of BPs

The resin tends to distribute at the top region of the rib in the composite BPs, which may impact the intrinsic TP thermal conductivity. To evaluate the effect of resin distribution on the thermal property of composite BPs, the thermal conductivity is calculated following the Equation (1),

(1) $$K=\alpha \cdot \rho \cdot C_{v}$$

where *K*, α, ρ, and *C*_v_ are thermal conductivity, thermal diffusivity, density, and specific heat capacity, respectively.

#### 3.2.1. Density of Composite BPs

Based on the microstructure characterization, the thermal-electrical properties of composite BPs were further tested. Table 2 shows the weight ratio of resin in a graphite matrix, theoretical density, true density, the corresponding porosity, and thickness of BPs. Specifically, the weight ratio of resin in the composites varies slightly under high molding pressure combined with increasing impregnation pressure (38%–42%). This indicates that the pores in the expanded graphite matrix were effectively removed under high molding pressure, and the effect of increasing the impregnation pressure on the weight rate of resin in the graphite matrix could somehow be neglected. The corresponding theoretical density of composite BPs, calculated according to the mixture rule, is similar (1.57–1.62 g/cm^3^) and is much smaller than the density of graphite (2.33 g/cm^3^). The true density of BPs, tested according to the Archimedes method (test sample > 2 g), ranges from 1.54 to 1.59 g/cm^3^, which is higher than the porous graphite matrix (1.28 g/cm^3^). This indicates that the pores in the porous graphite matrix have been effectively saturated by resin, which can also be confirmed from the corresponding relative density. In addition, the thickness of composite BPs was tested and ranges around 1 mm. The true density of composite BPs in this work is smaller than those reported in literature [21], which helps to enhance the power density of PEMFCs. The true density of composite BPs was used for calculating the thermal conductivity in the following section.

#### 3.2.2. Thermal Diffusivity of Composite BPs

The TP thermal diffusivity of composite BPs is tested based on the transient thermal conduction (φ12.7 × 1 mm). To mimic the fuel cell working temperature condition, the TP thermal diffusivity of composite BPs at different temperatures (25 °C, 50 °C, and 80 °C) were tested to evaluate the effect of temperature on the thermal performance.

**Figure 5:**
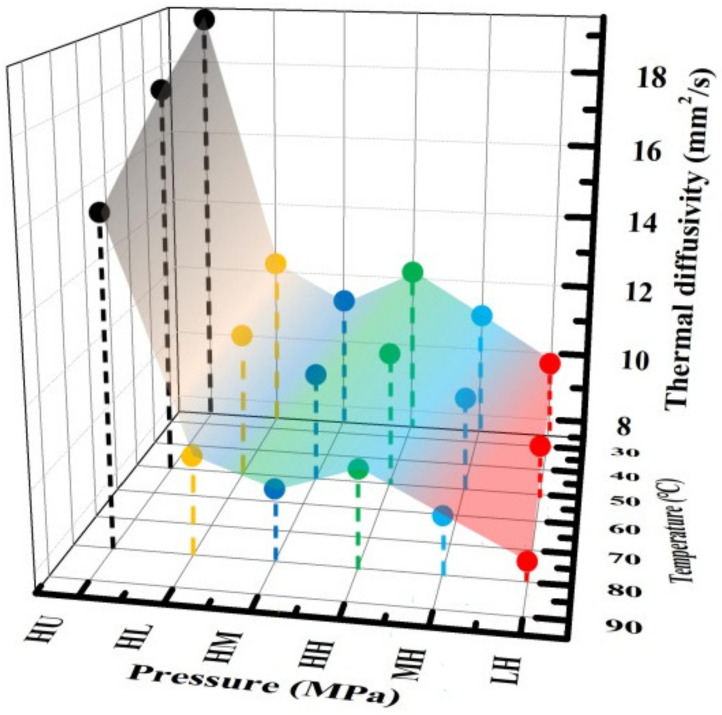
Through-Plane (TP) thermal diffusivity of composite BPs at different temperatures.

Figure 5 shows the TP thermal diffusivity of composite BPs at different temperatures and different pressures. The front *X*-axis represents different combination of molding pressure and impregnation pressure; the right side *X*-axis represents the test temperature, and the *Y*-axis represents the thermal diffusivity. Generally, the TP thermal diffusivity of the composite BPs decreases gradually with the increase of temperature, which could be mainly attributed to the increased perturbation of phonons under increasing temperatures. For composite BPs under high molding pressure, the TP thermal diffusivity decreases and then increases at each temperature with increasing impregnation pressure and are much smaller than the HU-BP. The smaller TP thermal diffusivity in the HM-BP can be mainly attributed to the low relative density, and the residual pore defects may impact the heat transfer path between the interlayer of graphite sheets. In addition, the decreased TP thermal diffusivity in the composite BPs confirms that the impregnation of resin deteriorates the crystal structures of the graphite matrix (displayed in XRD and Raman spectra) and further impacts the thermal/electrical conduction in the composite BPs. The TP thermal diffusivity increases at each temperature for composite BPs under increasing molding pressure combined with high impregnation pressure. This is because the interlayer spacing between the graphite sheets can be effectively densified by the increasing molding pressure, which facilitates the heat transfer in the TP direction.

**Figure 6:**
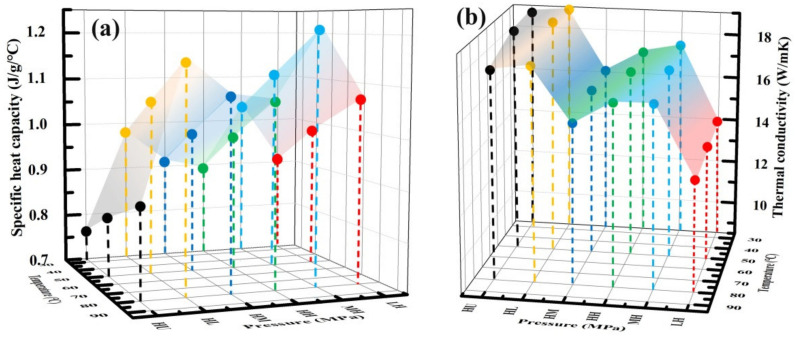
Thermal properties of composite BPs. (**a**) Specific heat capacity and (**b**) TP thermal conductivity of composite BPs under different temperatures.

Figure 6a shows the variation of specific heat capacity of composite BPs at different temperatures. The front *X*-axis represents different combination of molding pressure and impregnation pressure; the left side *X*-axis represents the test temperature, and the *Y*-axis represents the heat capacity. The specific heat capacity increases slightly with increasing temperature for composite BPs (0.76–1.21 J/g/°C), which is similar to that of the expanded graphite (HU-BP) and the variation trend reported in literature [22]. In addition, the specific heat capacity is remarkably higher for the composite BPs compared to the HU-BP, which could be due to the impregnation of resin in the expanded graphite matrix.

Figure 6b shows the TP thermal conductivity of composite BPs at different temperatures and different pressures obtained from Equation (1). The front *X*-axis represents different combination of molding pressure and impregnation pressure; the right side *X*-axis represents the test temperature, and the *Y*-axis represents the thermal conductivity. The TP thermal conductivity decreases gradually with increasing temperature, which is similar to the variation in the thermal diffusivity and could be due to the increased perturbation of phonons under increasing temperatures. The TP thermal conductivity of the composite BPs decreased slightly from 18.8 to 17 W/mK (variation ratio, 11%) with increasing impregnation pressure at 25 °C, which could be due to the degraded crystal structure of graphite induced by the impregnation of resin. For composite BPs at high impregnation pressure, the TP thermal conductivity increases with increasing molding pressure. Overall, the TP thermal conductivity is higher for composite BPs at high molding pressure than those at high impregnation pressure. This can be attributed to the crystal structure deterioration and expanded interlayer spacing induced by the impregnation of resin into the graphite matrix. Consequently, the key factor in improving the thermal and electrical conductivity of composite BPs is maintaining the lamellar structure of the graphite matrix that could provide a continuous network for heat and electron conductions [23]. In addition, it should be noted that the intact crystal structure of graphite should be kept from deterioration by the impregnation of resin, which enables high heat and electron conduction capacities.

### 3.3. Electrical Property of BPs

**Figure 7:**
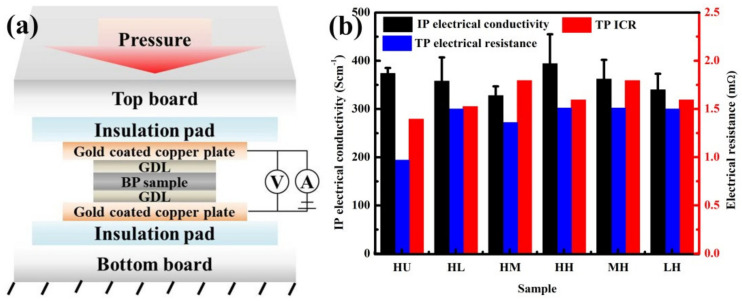
Electrical properties. (**a**) Illustration on TP electrical resistance and overall Interfacial Contact Resistance (ICR) test, and (**b**) IP electrical conductivity, TP electrical resistance, and overall ICR of composite BPs.

The cell voltage of PEMFC is greatly affected by the Ohm loss induced by the interfacial electrical contact resistance between the BP and GDL. The overall ICR, intrinsic TP electrical resistance, and IP electrical conductivity, which are influenced by the fabrication process, are tested to evaluate the effects of molding and impregnation pressures on the electrical performance of composite BPs. Figure 7a shows the illustrations on ICR between the composite BP and GDL as well as the intrinsic TP electrical resistance test of composite BPs. The size of tested BP is set as 50 × 50 × 1 mm. To mimic the stack assembly under compaction, the BP sample was embedded between the GDLs and compacted under the applied pressure with a maximum value of 3 MPa. The electrical resistance was obtained by recording the value of voltage and current at different pressure.

Figure 7b shows that the IP electrical conductivity of composite BP is comparable to the HU-BP and ranges at 350–400 S cm^−1^ (variation ratio, 14%) with the maximum value displayed in the HH-BP. Notably, the IP electrical conductivity increases gradually with increasing molding pressure combined with high impregnation pressure. The IP electrical conductivity obtained in this work is much higher than that reported in literature [24,25]. The high IP electrical conductivity could be mainly attributed to the well-reserved network of graphite in the composite BP, which acts as electron conduction path to enhance the electrical conductivity in the IP direction. As mentioned in the thermal conductivity section, the low IP electrical conductivity displayed in the HM-BP could be attributed to the high porosity.

The overall ICR (red color), which consists of the intrinsic TP electrical resistance and ICR between the BP and GDL, is slightly higher for the composite BP than the HU-BP. The TP ICR of the composite BP (0.5–5 mΩ·cm^2^) in this work is much smaller than the TP ICR reported in literature [26]. The intrinsic TP electrical resistance is slightly higher for the composite BP than the HU-BP, which could be attributed to the deteriorated crystal structure induced by the impregnation of resin. Nevertheless, the minimum ICR displayed in the HL-BP could be attributed to the densified interlayer spacing under high molding pressure, in which the continuous lamellar structure of graphite is simultaneously well-reserved under low impregnation pressure. The well-reserved electrical property can facilitate the electron conductions in the PEMFCs, which contributes to the power density improvement.

### 3.4. Compressive Strength of BPs

A bipolar plate with satisfied compression performance is required for keeping the assembly of fuel cell stacks from gas leakage and deformation failure. The effect of molding and impregnation pressures on the compressive strength of composite BPs were evaluated using the compressive test with the following parameters: the overall size is 30 ×30 × 1 mm; the maximum load is 4 × 10^4^ N, and the loading rate is 0.5 mm/min. It should be noted that the maximum compressive strength of the rib is defined as the compressive strength of the composite BP. Since the failure of the rib will lead to the destruction of the flow field, which further affects the electron, heat, and mass transfer of the fuel cell.

**Figure 8:**
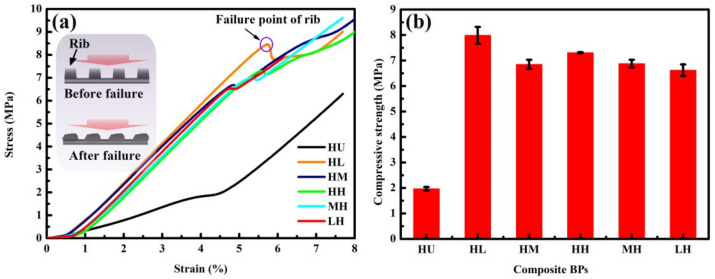
Mechanical performance of composite BPs. (**a**) Compressive stress-strain curves of composite BPs and the (**b**) corresponding compressive strength.

Figure 8 shows the compressive performance of composite BPs. Figure 8a shows the stress–strain curves of composite BPs, in which the stress increases linearly with the increase of strain before the point of rib failure. The stress reaches the peak value (marked with a purple circle) and starts to decrease as the rib is broken (schematically illustrated in the inset of Figure 6a). Another increase in the stress occurs when the channel is under compaction after the failure of the rib. It is indicative that the peak value is higher for the HL-BP than that of the other composite BPs, which could be due to the well-reserved network of graphite in the composite BPs. The continuous network of graphite in the composite BPs act as a load transfer bridge, which could effectively enhance the deformation resistance of the composite BPs. Notably, the compressive strength is much higher for the composite BPs than the HU-BP, which indicates that the mechanical property is effectively enhanced by the impregnation of resin.

Figure 8b shows the compressive strength of composite BPs, which is obtained by averaging three valid test data. The compressive strength of composite BPs is much high than the HU-BP (2 MPa) and ranges at 6.5–8 MPa. The compressive strength of HL-BP (~8 MPa) is much higher than other composite BPs. This can be due to the well-reserved lamellar graphite structure under high molding pressure that acts as a continuous network in the composite BP with resin filled in the pores under low impregnation pressure. The continuous network of graphite acts as effective load transfer bridge, which contributes to the strengthening effect in composite BPs as reported in the previous work [27]. In contrast, the lamellar network of graphite in the HH-BP may be destructed by the resin under high impregnation pressure, which leads to a decreased compressive strength.

The compressive strength increases gradually for composite BPs under increasing molding pressure combined with high impregnation pressure. The resin is saturated in the compacted lamellar structure of graphite under high impregnation pressure, and the increase of molding pressure can promote the compactness of the graphite matrix. The compressive strength is slightly higher for composite BPs under high molding pressure than those under high impregnation pressure except the HM-BP. This indicates that the continuous network of graphite matrix formed by the high molding pressure dominates the compressive strength of composite BPs.

**Figure 9:**
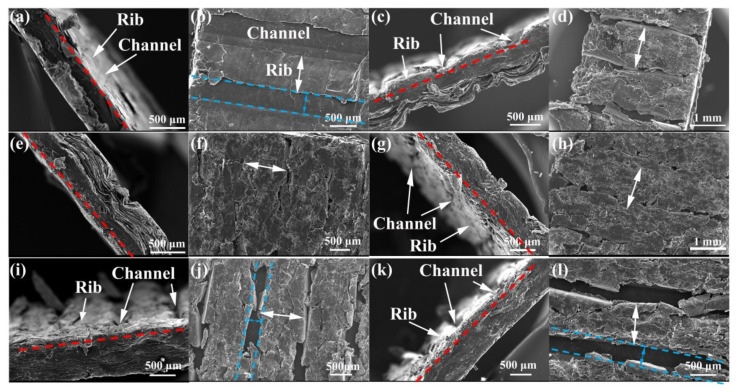
Compressive failure morphology of composite BPs. Cross-section morphology of (**a**) HU-BP, (**c**) HL-BP, (**e**) HM-BP, (**g**) HH-BP, (**i**) MH-BP, and (**k**) LH-BP; the corresponding in-plane surface morphology of (**b**) HU-BP, (**d**) HL-BP, (**f**) HM-BP, (**h**) HH-BP, (**j**) MH-BP, and (**l**) LH-BP.

Figure 9 shows the failure morphology of composite BPs under compression test. Figure 9a shows the cross-section morphology of HU-BP after failure. The red dotted line shows the surface faced to compression, compared to the original morphology of ribs in the HU-BP, the morphology of ribs after compaction shows that the rib was almost the same level of the channel bottom. Figure 9b shows the corresponding in-plane morphology, it is indicative that the rib is vertically compressed without overflowing to the channel region (blue dotted lines and arrow). Figure 9c shows the cross-section morphology of HL-BP after failure, compared to the original morphology of ribs in HL-BP, the rib is severely compressed, and the residual rib is slightly higher (red dotted line) than the channel bottom after failure, which can be mainly due to the enhanced deformation resistance induced by the impregnated resin in the top region of rib. While the corresponding in-plane morphology in Figure 9d shows that the broken rib overflew to the adjacent channels after failure, indicating the compressive deformation resistance of the rib is effectively enhanced by impregnating resin. Similar failure morphologies, i.e., the slightly higher rib (red dotted lines) and overflow of broken ribs (white arrows) are also displayed in the HM-BP and HH-BP, as shown in Figure 9e–h. By contrast, for composite BPs under low and medium molding pressures combined with high impregnation pressure, as shown in Figure 9i–l, the height of the rib is partly reserved after compressive failure and is much higher than the channel bottom (red dotted lines). Meanwhile, the channel regions were partly reserved since the fractured ribs were not severely overflown to the channel regions (blue dotted arrow and lines).

In general, the compressive strength and the moldability of rib in the composite BPs could be significantly enhanced by impregnating the resin. While it should be noted that the rib tends to be more brittle and the corresponding compressive strength is decreased for composite BPs under high impregnation pressure than those under high molding pressure. This could be attributed to the higher weight ratio of resin in the composite BPs under high impregnation pressure. The thermoset resin promotes the moldability of composite BPs and enhances the mechanical strength to some extent. While the impregnation of resin under high pressure may destruct the continuous lamellar structure of the graphite matrix, hence impacting the compressive strength.

**Figure 10:**
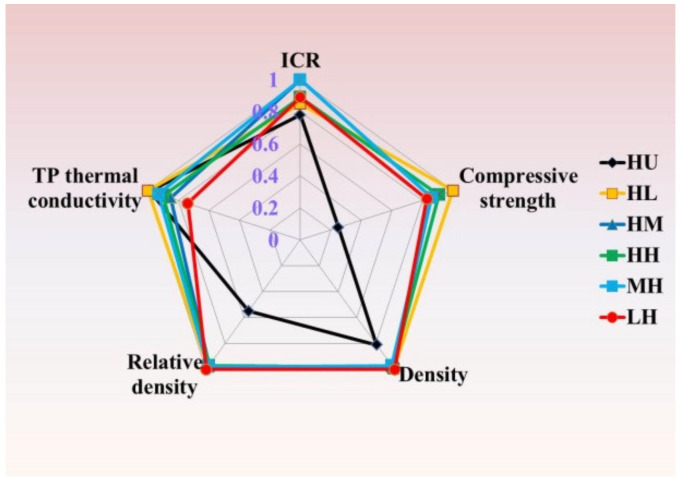
Thermal-electrical-mechanical property of composite BPs.

Figure 10 shows the comparison of thermal-electrical-mechanical properties between composite BPs under different molding and impregnation pressures, which includes density, porosity, thermal conductivity, ICR, and compressive strength. The relative density and compressive strength of graphite-based composite BPs are greatly enhanced by the impregnation of resin, which contributes to the gas tightness and durability of PEMFC stacks. The thermal and electrical conductivities of expanded graphite bulk are effectively reserved in the composite BPs under high molding pressure. The maximum TP thermal conductivity, compressive strength, and minimum ICR are collectively displayed in the HL-BP, which indicated that the thermal-electrical-mechanical properties of composite BP could be synergetically improved by the coupled manipulations of molding and impregnation pressures.

## 4. Conclusions

In this paper, graphite-resin composite BPs were designed and fabricated using different molding and impregnation pressures. The impacts of the molding and impregnation pressures on the microstructure and properties were investigated, respectively. The results show that the resin tends to distribute at the top regions of the rib under high impregnation pressure. The TP thermal conductivity and electrical conductivity of the composite BPs were well reserved in the composite BP and are comparable to that of the expanded graphite (HU-BP). The compressive strength of composite BPs was effectively enhanced by impregnating resin, which is considered to enhance the gas tightness and mechanical performance. The collectively enhanced TP thermal conductivity, compression strength, and minimum ICR in the HL-BP through the coupled manipulation of the high molding and low impregnation pressures could be attributed to the well-reserved continuous network of graphite in the composite BPs. Graphite filled with resin composite BPs with low density, high thermal, electrical, and mechanical properties could contribute to the power density and durability improvements of PEMFCs. The findings in this paper may offer an effective approach that could collectively optimize the thermal-electrical-mechanical properties of graphite-based composite BPs through the coupled manipulation of molding and impregnation pressures, which is expected to enhance the power density and durability of PEMFCs in the 80 kW level.

## Figures and Tables

**Table 1:** Different combination of molding and impregnation pressures for fabricating composite bipolar plates (BPs).

	Impregnation	High	Medium	Low
Molding				
High		HH	HM	HL
Medium		MH	×	×
Low		LH	×	×

**Table 2:** Weight ratio of resin, theoretical density, true density, porosity, and thickness of BBPs.

	HU	HL	HM	HH	MH	LH
Weight ratio of resin (wt.%)	/	38.7	38.2	39.1	41.5	40.8
Theoretical density (g/cm^3^)	2.33	1.61	1.62	1.60	1.57	1.58
True density (g/cm^3^)	1.28	1.57	1.56	1.56	1.54	1.59
Relative density (%)	55%	97.5%	96.5%	97.3%	97.8%	99.8%
Thickness (mm)	0.895	0.901	0.931	0.921	0.971	0.952

## Data Availability

The data presented in this study are available on request from the corresponding author.
